# MCL-DTI: using drug multimodal information and bi-directional cross-attention learning method for predicting drug–target interaction

**DOI:** 10.1186/s12859-023-05447-1

**Published:** 2023-08-26

**Authors:** Ying Qian, Xinyi Li, Jian Wu, Qian Zhang

**Affiliations:** Shanghai Frontiers Science Center of Molecule Intelligent Syntheses, School of Computer Science and Technology, East China Normal University, North Zhongshan Road, Shanghai, 200062 China

**Keywords:** Drug–target interaction, Deep learning, Multimodal information, Multi-head self-attention mechanism, Cross-attention mechanism

## Abstract

**Background:**

Prediction of drug–target interaction (DTI) is an essential step for drug discovery and drug reposition. Traditional methods are mostly time-consuming and labor-intensive, and deep learning-based methods address these limitations and are applied to engineering. Most of the current deep learning methods employ representation learning of unimodal information such as SMILES sequences, molecular graphs, or molecular images of drugs. In addition, most methods focus on feature extraction from drug and target alone without fusion learning from drug–target interacting parties, which may lead to insufficient feature representation.

**Motivation:**

In order to capture more comprehensive drug features, we utilize both molecular image and chemical features of drugs. The image of the drug mainly has the structural information and spatial features of the drug, while the chemical information includes its functions and properties, which can complement each other, making drug representation more effective and complete. Meanwhile, to enhance the interactive feature learning of drug and target, we introduce a bidirectional multi-head attention mechanism to improve the performance of DTI.

**Results:**

To enhance feature learning between drugs and targets, we propose a novel model based on deep learning for DTI task called MCL-DTI which uses multimodal information of drug and learn the representation of drug–target interaction for drug–target prediction. In order to further explore a more comprehensive representation of drug features, this paper first exploits two multimodal information of drugs, molecular image and chemical text, to represent the drug. We also introduce to use bi-rectional multi-head corss attention (MCA) method to learn the interrelationships between drugs and targets. Thus, we build two decoders, which include an multi-head self attention (MSA) block and an MCA block, for cross-information learning. We use a decoder for the drug and target separately to obtain the interaction feature maps. Finally, we feed these feature maps generated by decoders into a fusion block for feature extraction and output the prediction results.

**Conclusions:**

MCL-DTI achieves the best results in all the three datasets: Human, *C. elegans* and Davis, including the balanced datasets and an unbalanced dataset. The results on the drug–drug interaction (DDI) task show that MCL-DTI has a strong generalization capability and can be easily applied to other tasks.

## Introduction

Prediction of drug–target interactions (DTIs) is an essential step for drug discovery (i.e., to find new candidate drugs) and drug reposition (i.e., to find new indications for existing drugs). Drugs play an important role in the human body by interacting with multiple targets [1]. Proteins represent an important type of targets whose function can be enhanced or inhibited by drugs to achieve phenotypic effects for clinical therapeutic purposes [2]. However, traditional experiments to obtain drug candidates through bioanalysis typically take 10–15 years and cost approximately 1 billion dollars from introducing the abstract concept to release into the market [3]. Large number of computational approaches are proposed for this task aim to mitigate the costs and risks of drug development.

Over the past decades, many computational methods have been widely applied to predict DTIs [4–8]. These computational methods can be mainly divided into three groups: docking-based methods [9, 10], ligand-based methods [11, 12], and chemogenomic-based methods [2, 4]. Docking-based methods cannot be applied if the 3D structure information for many target proteins is unknown. Ligand-based methods will not be suitable when the number of known ligands is limited or few. The chemogenomic-based methods overcome the limitations by utilizing the chemical and genomic information of drugs and targets that are available in many online public databases. Currently, machine learning and deep learning approaches are very popular. Several studies [7, 13–17] have summarized the progress of ML and DL methods in DTI prediction tasks. Traditional machine learning methods include network-based methods [18–22], clustering-based methods [23], kernel-based methods [24–28], and matrix factorization-based methods [29–33].

Deep learning approaches generally treat the DTI task as a binary classification task by first learning the embedded representations of the drug and target separately and then connecting them for prediction. In the DTI task, according to the representation of drugs and proteins, we can categorize the mainstream deep learning methods into three groups, sequence-based methods, graph-based methods, and image-based methods.

Sequence-based approaches are more common. DeepDTA [34] uses a convolutional neural network to learn drug and protein sequence features, DrugVQA [35] uses a bi-directional long-short time memory network (BiLSTM) for feature extraction of sequence information, and TransformerCPI [36] builds a transformer architecture with a self-attention mechanism. The main idea of these methods is to construct neural networks to learn useful information from drug and target sequences for DTI task. Moltrans [37] propose an innovative FCS (Frequent Subsequence Algorithm) algorithm to decompose protein and compound sequences. By employing an augmented transformer, they successfully capture the semantic characteristics of substructures from a large volume of unlabeled biomedical texts. DeepCDA [38] combines CNN and LSTM to encode protein and compound sequences, and proposes a bidirectional attention mechanism to encode the intensity of their interaction. In order to solve the problem of sampling test and training data from different distribution domains, DeepCDA [38] also utilizes adversarial domain adaptation methods to learn the feature encoder network in the test domain.

Graph-based neural networks have become a prominent approach for extracting abstract features of drug. The RDKIT toolkit can transform the drug into graph structures, enabling the application of graph neural networks (GNN) in the CPI task [39]. GraphCPI [40] and GraphDTA [41] adopts Graph Convolutional Networks (GCN) to perform convolutional operations on compound graph structures. LGDTI [42] is based on large-scale graph representation learning to predict DTI. Compared with the existing graph based neural network methods, LGDTI adopts a unique method to extract the potential graph features of drugs and targets in complex biological network by using two different graph representation learning methods. FuHLDR [43] is a novel graph representation learning model for drug repositioning, which effectively integrates high-level and low-level biological information. It provides a new solution for constructing heterogeneous information networks for DTI tasks to improve prediction accuracy.

Image-based methods were previously underappreciated. Image-based approach to extract useful features from molecular images of drugs. PWO-CPI [44] constructs a CNN model to learn the features in molecular images as the embedding representation of the drug and uses word2vec [45] model learn the protein sequences.

These methods only considered single modal information of the drug, such as SMILES sequences, molecular graphs or molecular images. Huang et al. [46] worked out to the conclusion that the richer the variety of modalities, the more accurate the estimation of the representation space with sufficient training data. In order to obtain more comprehensive features of drugs, some researchers also use both the sequence and graph structure of drugs to achieve DTI tasks, such as SSGraphCPI [47]. This method can obtain effective information from the two modalities of drugs, which can effectively improve the effectiveness. In the field of computer vision, multimodal techniques are also widely used for various tasks, such as visual Question Answering, Image Caption, Referring Expression Comprehension and Visual Dialogue [48, 49]. In tasks such as DTI and interaction prediction, few people consider the combination of drug images and other information. Therefore, we will further discuss whether fusing and enhancing multiple modal information have improved drug and target embedding representation. In addition, TripletMultiDTI [50] is also a new multimodal DTI method, which designs a new architecture that integrates multimodal knowledge to predict affinity labels. At the same time, it also proposes a new loss function based on the triplet loss, making the model perform better. TranSynergy [51] designs an enhanced deep learning model based on knowledge and self attention machine mechanism to predict collaborative drug combinations, effectively improving the performance and interpretability of collaborative drug combination prediction.

In our previous work PWO-CPI [44], we have shown that the features of drug image can be well used for the task of DTI. In addition, the information contained in a single image is not sufficient to fully characterize the drug. We want to introduce chemical properties that are valuable for understanding compounds. Therefore, we propose that combining the images of compounds and chemical features of drugs can lead to a more comprehensive abstract characterization of drugs, which can enhance the DTI results.

Cross-attention mechanisms are often used in image description generation, visual questioning and answering, where it can cross-learn features from multiple modalities. This cross-attention mechanism enhances the expressive power of feature representation by introducing an attention mechanism to dynamically adjust the association weights between multimodal features, thus realizing effective feature fusion and interaction. Therefore, this paper proposes to introduce the cross-attention mechanism into the learning of drugs and targets features, so that the cross-learning of the above two features can be realized to extract the correlation relationship between the two, which helps to improve the performance of the DTI task.

In general, the main contributions of this paper are as follows:

- In this study, we introduce a novel approach by integrating the multimodal information of compound images and chemical text information as input features for drugs. We can extract more comprehensive drug features from both modalities, which are effectively used for DTI tasks.
- An innovative method of bi-directional cross-attention learning is proposed. This bi-directional cross-attention learning mechanism can learn deeper semantic relationships between drugs and targets, capturing more useful interaction features to enhance DTI prediction effects.
- Improved predictive performance over state-of-the-art baselines on three public datasets with different scales. The DTI experimental results demonstrate the effectiveness of the method. The excellent results on the DDI task demonstrate the generalization of the method proposed in this paper.

## Method

### Overall workflow

**Fig. 1:**
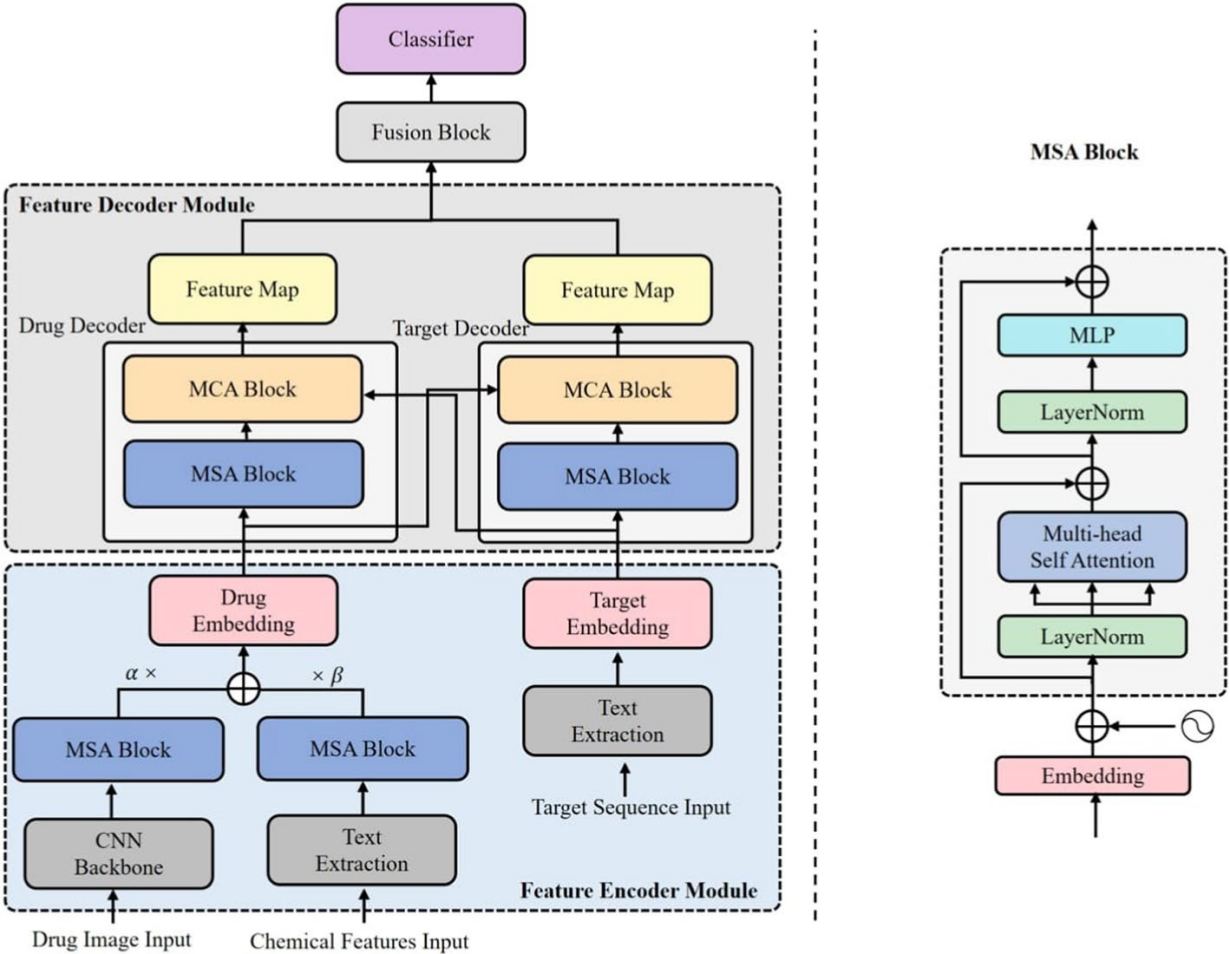
An overall architecture of MCL-DTI

DTI can be regarded as a classification problem, inputting drugs and targets into the model to predict where the two will interact with each other. If there is an interaction between the two, output 1, otherwise output 0. The method proposed in this paper inputs drug multimodal information as well as the FASTA sequence of the target into the model, and the predictive goal of the model is to output whether the two interact. The architecture of the MCL-DTI model is shown in Fig. 1. The whole model mainly consists of four modules: feature encoder module, feature decoder module, feature fusion module, and classifier. We use the Rdkit toolkit to obtain images and chemical features of drugs from SMILES sequences, used as multimodal representations of the drug. We input the multimodal representations of the drug and the sequence representation of the target into the feature extraction module, obtain the high-level abstract features of them respectively, and then feed them into the feature decoder module. The feature decoder module consists of independent drug decoder and target decoder, which are composed of MCA (Multi-head Cross Attention) Block and MSA (Multi-head Self Attention) Block. The feature decoder module can effectively decode the information of the drug and target as well as the interaction information between them. After the feature decoder, we send the two obtained feature maps to the feature fusion module, and then a classifier to get the final prediction result. We will describe each module in detail in the next few sections.

### Feature encoder module

**Fig. 2:**
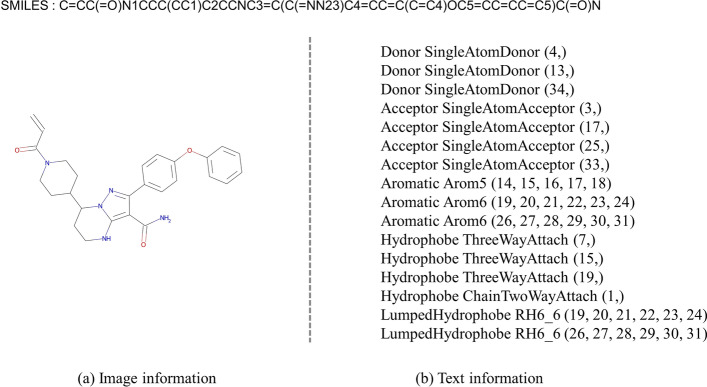
Multimodal information of drugs. **a** is the molecular image modal. **b** is the chemical text information modal

To better capture the drug features, we input the image and the chemical features text of the drug. For drug image, we construct a CNN backbone *Conv* similar to PWO-CPI [44]. This backbone contains convolution, batch normalization, activation and pooling layers. We first obtain the structural formula images of drugs from SMILES sequence by RDKit [52] software. These images show visual representations of molecule, as can be seen in Fig. 2a. We define the input image as $P\in R^{h \times h}$, where *h* denotes the size of image. The local feature map $x^v=Conv(P)$ of the image can be obtained by the constructed CNN backbone. Since CNN Block can only capture local information without considering global features, we build an MSA block to enhance semantic relations of features, and the specific flowchart of MSA is shown on the right side of Fig. 1. MSA block contains Layer Normalization (LN) [53] layers, multi-head self attention layer, MLP block and residual connections. Following prior works on transformers encoder in [54], we add a residual connection to the MSA computation. LN layers are applied before every block to normalize neuron nodes in the neural network. We pass the output $x^v=Conv(P)$ of the CNN Block through the MSA block to get the image feature of the drug, $X_{img}=MSA(x^v)$.

**Fig. 3:**
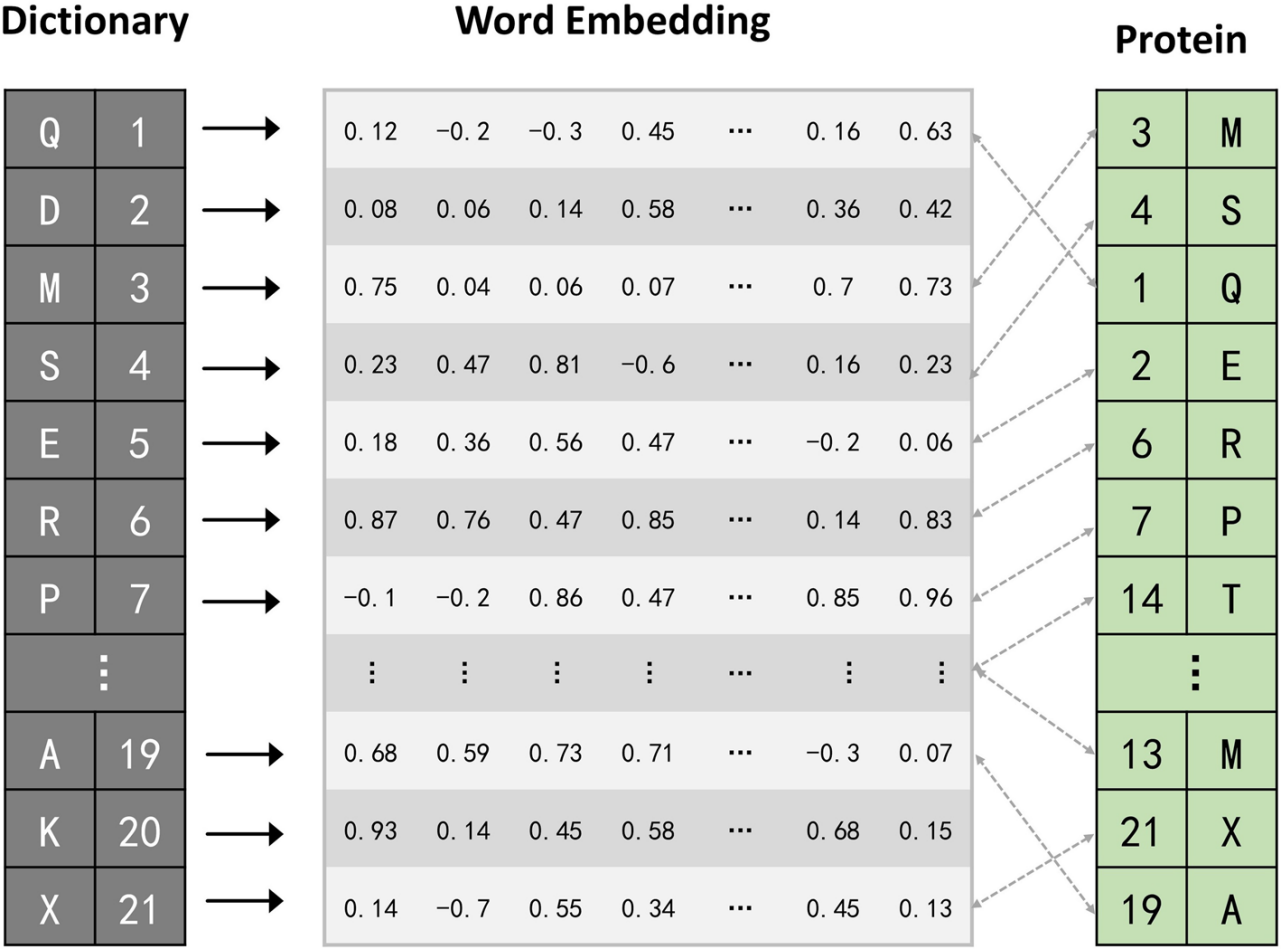
Method for protein sequence segmentation and embedding representation when k is 1

The chemical features are defined by a feature type and a feature family. A feature family is a general classification of features, such as hydrogen bond donors, aromaticity, etc., where pharmacophore matching is achieved based on the feature family [52]. Here we use a feature factory and choose feature family information, feature type information and feature corresponding atoms information as the chemical text information of the drug. We can obtain the chemical text information of the drug by Rdkit [52] software using the SMILES sequences, as can be seen in Fig. 2b. In order to extract features from drug text, we first use the $k-gram$ method to segment the text sequences by words. The text sequences are divided into phrases of length *k*, and build a dictionary to record the order in which the phrases appear. The numerical word order of the dictionary is used to replace the original words, and these numbers are embedded for representation. Figure 3 shows $k-gram$ method of protein sequence segmentation and embedding representation when *k* is 1. Similarly, we feed the embedding representation into an MSA module to obtain textual features of the compound, $X_{text}$.

We take the sum of $X_{img}$ and $X_{text}$ as the drug’s features, while we assign learnable weights $\lambda _1$ and $\lambda _2$ to them. A higher weight indicates that the modality has a large influence on the drug feature representation. The drug is encoded as $X_{drug}$:

1 $$\begin{aligned} X_{drug}=\lambda _1 X_{img}+\lambda _2X_{txt} \end{aligned}$$

For target sequence, we directly use its FASTA sequence as its text information. Similar to the chemical feature text of drug, we do the same for the FASTA sequence, first obtaining its embedding representation through k-gram, and then obtaining the abstract features of the target $X_{tgt}$ through an MSA module.

### Feature decoder module

**Fig. 4:**
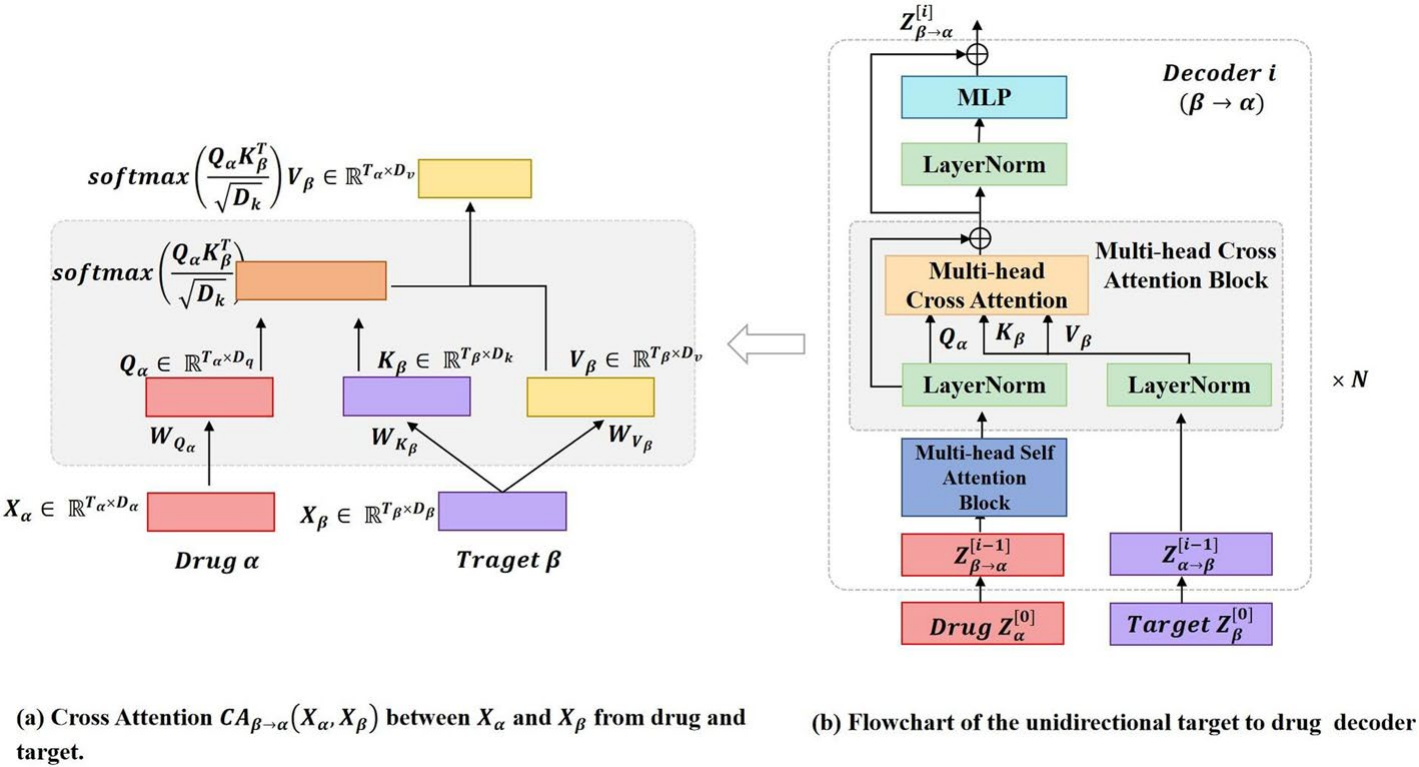
Architectural elements of a cross attention block between two time-seriers form drug $\alpha$ and target $\beta$

After encoding the drug and target features, we feed the obtained $X_{drug}$ and $X_{tgt}$ to the feature decoder module to learn the drug–target interaction information. As shown in Fig. 1, the feature decoder module consists of two decoders and each consists of an MSA block and MCA block. MCA block have the same LN layers, MLP blocks, residual connections with MSA layers. The main difference between MSA and MCA is the calculation process of attention output. The MSA block is designed to capture the internal relationships of the features themselves, and when computing the attention output, the *query*, *key*, and *value* are all obtained from the same feature through a linear matrix transformation. The MCA block, on the other hand, is designed to capture the interaction information between the drug and the target. Therefore, for the MCA block of the drug decoder, not only the drug features but also the target features need to be inputted. We perform matrix linear transformation on the input target features to get the *query* needed to compute the attention output, and perform linear transformation on the input target features to get the *key* and *value*. Figure 4 illustrates the computational process of MCA. The target decoder is similar. With the drug decoder and the target decoder, we send their respective features to each other in both directions for two-way cross learning, and finally get two feature maps, $Z_{drug \rightarrow target}$ and $Z_{target \rightarrow drug}$.

### Feature fusion module

The fusion block is used to receive the input feature maps $Z_{drug \rightarrow target}$ and $Z_{target \rightarrow drug}$ from two decoders. We concatenate both feature maps by channel dimension and feed it into the fusion block. Fusion block contains a 2D convolution network *Conv*2*D*, a 1D convolution network *Conv*1*D*, a MLP block *MLP* and a fully connected layer *FC*. We extract the concatenated feature maps by convolution layers and finally feed them into FC layer to obtain the final prediction result *P*, this calculation can be expressed as:

2 $$\begin{aligned} P=FC(MLP(Conv1D(Conv2D(Z_{drug \rightarrow target};Z_{target \rightarrow drug})))) \end{aligned}$$

where *Z* represents the feature map generated by decoder and ;  denotes the concatenate operation.

### Classifier

We use cross-entropy as loss function specifically as follows:

3 $$\begin{aligned} Loss=-\frac{1}{N}\sum _{n=1}^{N}(y_n log(P_n)+(1-y_n)log(1-P_n)) \end{aligned}$$

where *N* denotes the total number of samples, and $y_n$ represents the true label. When model training, w choose the Adam [55] optimization algorithm as the optimizer of the model.

## Experiment

In this section we present experimental comparisons of MCL-DTI with other state-of-the-art methods.

### Experimental setup

#### Dataset

**Table 1:** DTI dataset statistics

Datasets	Drugs	Targets	Samples	Pos Samples
Human	2496	1919	6184	3364
*C. elegans*	1716	1856	7509	3892
Davis	64	379	10439	1428

In this work, we choose three DTI public datasets for experiments including Human [56], *C. elegans* [56] and Davis [57]. See Table 1 for specific drug and target statistics. Human and *C. elegans* are both positive and negative sample balanced datasets. Their positive samples are obtained from the highest confidence biochemical databases: DrugBank [58] database and matador [59] database [56]. Davis contains 64 different drugs and 379 targets. In Davis, DTI pairs which have $k_d$ values < 30 units are considered positive [57]. Human and *C. elegans* datasets are divided into 8:1:1 ratio according to train set, valid set and test set when conducting the experiments. Davis dataset division is followed by MolTrans [37]. In addition, we use the Biosnap [60] for DDI task which is to predict the interaction between drug and drug. Biosnap contains 9,648 drugs and 81,194 samples with 50.5$\%$ of positive samples.

**Table 2:** Performance comparison

Dataset	Method	ROC–AUC	PR–AUC	Recall
Human	GNN-CPI [39]	0.974 ± 0.004	0.973 ± 0.005	0.953 ± 0.019
	DeepDTA [34]	0.953 ± 0.002	0.981 ± 0.003	0.946 ± 0.024
	DeepConv-DTI [61]	0.985 ± 0.001	0.982 ± 0.001	0.963 ± 0.002
	TransformerCPI [36]	0.971 ± 0.002	0.973 ± 0.002	0.942 ± 0.004
	PWO-CPI [44]	0.982 ± 0.003	0.980 ± 0.002	0.962 ± 0.001
	MolTrans [37]	0.978 ± 0.002	0.978 ± 0.001	0.933 ± 0.003
	MCL-DTI	0.987 ± 0.001	0.989 ± 0.001	0.961 ± 0.002
*C. elegans*	GNN-CPI [39]	0.978 ± 0.002	0.975 ± 0.005	0.949 ± 0.003
	DeepDTA [34]	0.987 ± 0.001	0.990 ± 0.002	0.964 ± 0.011
	DeepConv-DTI [61]	0.980 ± 0.002	0.981 ± 0.001	0.937 ± 0.003
	TransformerCPI [36]	0.985 ± 0.001	0.985 ± 0.002	0.952 ± 0.002
	PWO-CPI [44]	0.979 ± 0.002	0.978 ± 0.003	0.933 ± 0.003
	MolTrans [37]	0.985 ± 0.001	0.984 ± 0.002	0.962 ± 0.001
	MCL-DTI	0.992 ± 0.001	0.994 ± 0.001	0.959 ± 0.002
Davis	GNN-CPI [39]	0.840 ± 0.012	0.269 ± 0.020	0.696 ± 0.047
	DeepDTA [34]	0.860 ± 0.002	0.238 ± 0.001	0.818 ± 0.003
	DeepConv-DTI [61]	0.822 ± 0.003	0.192 ± 0.005	0.905 ± 0.004
	TransformerCPI [36]	0.841 ± 0.001	0.227 ± 0.003	0.842 ± 0.004
	PWO-CPI [44]	0.835 ± 0.004	0.188 ± 0.004	0.798 ± 0.003
	MolTrans [37]	0.907 ± 0.002	0.404 ± 0.016	0.800 ± 0.022
	MCL-DTI	0.922 ± 0.002	0.492 ± 0.002	0.895 ± 0.003

#### Metrics

In this work, we use ROC–AUC (area under the receiver operating characteristic curve), PR–AUC (area under the precision-recall curve) and recall as metrics to measure the prediction performance. The ROC–AUC is the main metric we use to evaluate all methods. The ROC–AUC curve takes into account both positive and negative examples and can effectively evaluate the overall performance of the model. The PR–AUC is more focused on positive examples, especially in data with unbalanced categories, and the value of PR–AUC is more indicative of the robustness of the model. Recall values indicate the percentage of samples predicted to be truly positive in the positive class. Recall provides good feedback on the model’s ability to learn for positive samples. The data for all results are expressed as the mean of the results and their standard deviation.

#### Experiment settings

The implementation of our method is based on Pytorch [62]. Each experiment is run for 100 epochs. For training, we use a server with i7 10700f, 32GB RAM and RTX 3090 GPU. For the selection of hyperparameters, we used the grid search method. The learning rate is searched in the range [1e−1, 1e−2, 1e−3, 1e−4, 1e−5], the learning rate decay coefficient is searched in the range [0.5, 0.6, 0.7, 0.8, 0.9], the batch size is searched in the range [32,64,128,256], the dropout rate is searched in the range [0.1,0.2,0.3,0.4,0.5]. We first use the grid search method to determine the learning rate and batch size, then fix the values of both, and then choose the dropout rate and learning rate decay coefficient. Through experiments, we choose the learning rate, learning rate decay coefficient, dropout rate, and batch size as 1e−3, 0.8, 0.1 and 128, respectively.

### DTI experiment

*Baseline*.When choosing the comparative models, we mainly consider from three perspectives: Firstly, we chose representative and state-of-the-art methods, including DeepDTA [34], TransformerCPI [36], and MolTrans [37], to validate the competitiveness of our model. These methods are widely recognized and frequently used as benchmarks. Secondly, to assess the effectiveness of image-based methods, we included GNN-CPI [39] and TransformerCPI [36], which are typical examples of sequence-based and graph-based approaches. Thirdly, as the MCL-DTI model is an extension of our team’s previous work, it was essential to include our previous model, PWO-CPI [44], for comparison. At last, We compare MCL-DTI with the following methods:

- GNN-CPI [39] uses molecular graph as drug representation and applies GNN for feature learning of embedded representation. They concatenate the outputs of the two neural networks for compound-protein interaction prediction. We follow the same hyperparameter setting described in this paper.
- DeepDTA [34] applies CNN to two original extraction of local residual patterns using SMILES and protein sequences. The task of DeepDTA is to predict binding affinity values. We add a sigmoid activation function at the end of the model to turn it into a binary task and we set the same hyperparameters to ensure fairness.
- DeepConv-DTI [61] uses CNN and global max pooling layers to extract local features of different lengths in protein sequences and applies the fully connected layer on drug fingerprint ECFP4. We obtain the same drug fingerprint ECFP4 and set the same hyperparameters for experimental comparison.
- TransformerCPI [36] uses the atomic information of the drug and distance matrix as a representation of the drug and a learned representation of the protein features by wod2vec [45]. They construct a decoder with a self-attention mechanism to learn the features of compounds and proteins.
- PWO-CPI [44] first uses drug images as molecular features. They use CNN to learn local information of drug images and apply word2vec [45] to encode protein sequences. Here, we use the same drug molecule images to represent the drugs and set the same hyperparameters for the experiments.
- MolTrans [37] builds a large corpus and extracts the most commonly used molecular fragments. The numbers are used to replace the original characters and embedding of these numbers is used for feature learning. It conducts extensive experiment on different datasets and is the SOTA method and this is also our main method of comparison.

To ensure the fairness of the experiments, we conduct experiments for other methods on the same dataset and use the same hyperparameter settings as in the original paper. The error between the reproduced results and the original results is acceptable. We use the cross-validation strategy and conduct five experiments for each method, and the final experimental results are shown in Table 2.

For the two balanced datasets Human and *C. elegans*, the current deep learning methods can achieve relatively promising performance. MCL-DTI achieves the best results and exceed our previous work PWO-CPI in all metrics. PWO-CPI only uses images of drugs and does not perform the operation of feature fusion. These experimental results demonstrate that MCL-DTI can effectively conduct feature learning on balanced datasets.

In addition, the deep learning methods for experiments on Davis dataset failed to achieve satisfactory results, especially in terms of PR–AUC values. In the test set of the Davis dataset, the ratio of positive to negative samples is 1:19, which tests the model’s ability to learn the full range of sample features under the same learning environment. Compared to MolTrans as SOTA method, MCL-DTI improved by 0.014, 0.073 and 0.069 for three metrics, respectively.

In summary, these deep learning methods all utilize only single modal information about the drug molecule such as molecular graph, SMILES sequence information and molecular image. MCL-DTI utilizes multimodal information, molecular images and chemical text information, so that it can provide more comprehensive information about drug. Results on both balanced and unbalanced datasets show the competitive performance of MCL-DTI.

Our excellent prediction results can be explained from the following perspectives:

1. From the biological perspective, the structure of a molecules determines their properties. The structural characteristics of molecules can be intuitively displayed in their images, and deep learning models have excellent performance in extracting spatial structural features of images. Therefore, integrating representations from molecules images can provide a more comprehensive understanding of the biological characteristics of these molecules.
2. Chemical characteristics provides valuable information about compounds’ properties. These characteristics, such as molecular weight, polarity, or functional groups, are very relevant to the interaction between compounds and proteins. By incorporating chemical characteristics, the model can learn to recognize and exploit these properties, leading to more accurate predictions of compound-protein interactions.
3. Integrating image features with the chemical properties of compounds at an advanced semantic level can better characterize the biological characteristics of compounds. The use of a multi-head cross-attention mechanism allows the model to learn the relationship between drugs (compounds) and targets (proteins) in a more sophisticated manner. This mechanism enables the model to focus on different aspects of the compounds and proteins simultaneously, capturing their intricate interactions. By learning the complex relationships between compounds and proteins, the model can better understand the underlying biological mechanisms and predict their interactions more accurately.

**Table 3:** Results on the Biosnap dataset in the DDI task

Dataset	Method	ROC–AUC	PR–AUC	F1
Biosnap	LR	0.802 ± 0.001	0.779 ± 0.001	0.741 ± 0.002
	Nat.Port	0.853 ± 0.001	0.848 ± 0.001	0.714 ± 0.001
	Mol2Vec	0.879 ± 0.006	0.861 ± 0.005	0.798 ± 0.007
	MolVAE	0.892 ± 0.009	0.877 ± 0.009	0.788 ± 0.033
	DeepDDI	0.886 ± 0.007	0.871 ± 0.007	0.817 ± 0.007
	Caster	0.910 ± 0.005	0.887 ± 0.008	0.843 ± 0.005
	MCL-DDI	0.996 ± 0.001	0.994 ± 0.001	0.986 ± 0.002

### DDI experiment

To further validate the learning ability of drug multimodality and MCA mechanism, we conduct experiments for DDI task. We use the same method as MCL-DTI for the embedding representation of drug. After obtaining the two drug embedding representations, these embedidng feature maps are fed into the same decoders as MCL-DTI to learn the interaction between different drugs respectively. Finally the prediction results are also obtained by a fusion block. We name this model for DDI tasks as MCL-DDI. Here we set ROC–AUC, PR–AUC and F1 values as indicators on Biosnap [60] dataset. Methods with which we have conducted experimental comparisons include LR [63], Nat.Prot [64], Mol2Vec [65], MoVAE, DeepDDI [66] and Caster [60].

The results of the DDI experiments are shown in Table 3. We find that MCL-DDI far exceeds the previous work in three different metrics. The performance of the model can indeed be effectively improved by multimodal and cross-attention learning of drugs. This also means that our model has strong generalization and is more suitable than previous methods for the prediction of both interactions.

**Table 4:** Results of ablation experiments on Human and Davis datasets

Dataset	Method	ROC–AUC	PR–AUC	Recall
Human	MCL-DTI	0.987	0.989	0.961
	Image + SMILES	0.983	0.982	0.926
	Text	0.963	0.966	0.910
	Image	0.983	0.985	0.935
	-MCA	0.984	0.984	0.897
	MCA, image	0.968	0.971	0.919
	MCA,text	0.980	0.982	0.945
Davis	MCL-DTI	0.922	0.492	0.895
	Iimage + SMILES	0.917	0.462	0.874
	Text	0.917	0.481	0.842
	Iimage	0.916	0.466	0.884
	MCA	0.914	0.471	0.835
	MCA, image	0.912	0.473	0.839
	MCA, text	0.913	0.453	0.839

**Table 5:** Ablation study on combining image modal and text modal

Dataset	λ_1_	λ_2_	ROC–AUC	PR–AUC	Recall
Human	$\lambda _1$	$\lambda _2$	0.990	0.992	0.961
	$\lambda _1$	1	0.984	0.983	0.942
	1	$\lambda _2$	0.982	0.984	0.945
	$\lambda _1$	$1-\lambda _1$	0.982	0.981	0.936
	1	1	0.987	0.989	0.955
	1	0	0.981	0.985	0.935
	0	1	0.968	0.968	0.920
Davis	$\lambda _1$	$\lambda _2$	0.922	0.492	0.895
	$\lambda _1$	1	0.917	0.475	0.877
	1	$\lambda _2$	0.918	0.467	0.861
	$\lambda _1$	$1-\lambda _1$	0.917	0.474	0.891
	1	1	0.916	0.486	0.874
	1	0	0.918	0.469	0.891
	0	1	0.916	0.487	0.856

### Ablation study

In this section, several ablation experiments are performed on the whole model to effectively represent the influence of each module on MCL-DTI. To better represent the robustness of each module of MCL-DTI, we conduct experiments on balanced and unbalanced datasets, i.e., Human and Davis.

- image + SMILES: we use the SMILES sequence of the drug as text information instead of the chemical text information
- Text: we use only the chemical text modal information as the drug embedding representation
- Image: we use only molecular image modal as the drug embedding representation.

- MCA: we remove the MCA block from drug and target decoders, so that only the MSA block remained in the decoder.
- MCA, image: we remove both MCA block and image modal.
- MCA, text: we remove both MCA block and text modal.

From the results in Table 4 we can see that in MCL-DTI as a complete model achieves the best results on both datasets.

The results for image + SMILES and image are similar, and we can see that the effect is not obvious when using SMILES sequences to enhance the features. This can indicate that the image may contain the information of SMILES sequences or more. It can be inferred from MCL-DTI and image + SMILES that the chemical text information of the drug contains different information from the SMIELES sequence. In the experiments of text and image, it can be seen that images play a more important role in the features of drug molecules. In addition, it can be observed from text that the information of the chemical text improves the model. The results from these experiments further demonstrate that the multimodal and cross-attention modules have latent capabilities for feature learning.

**Fig. 5:**
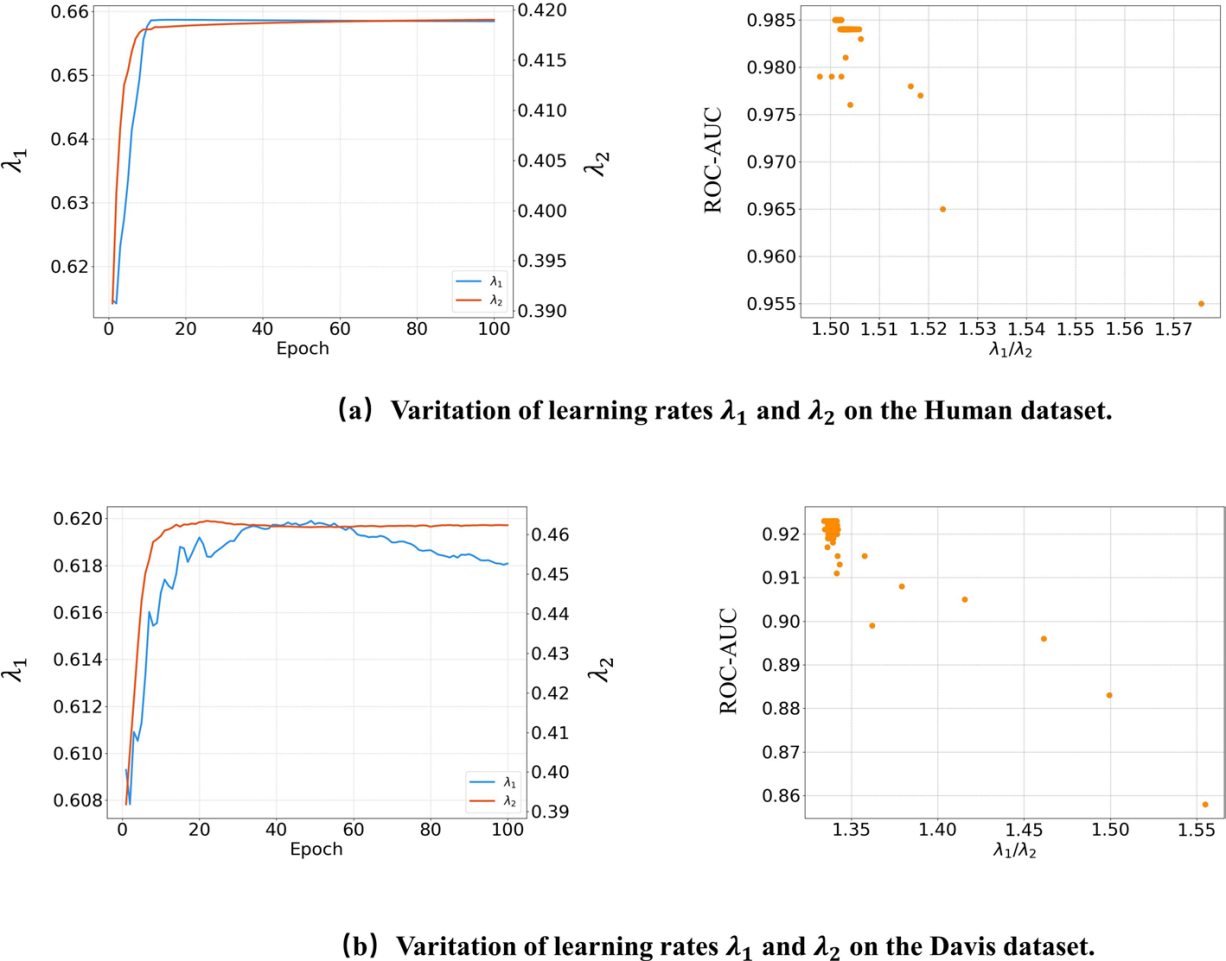
Variation of learnable variables $\lambda _1$ and $\lambda _2$ on the Human and Davis datasets. The process of change of scalars during the experiment and the learning ratio correspond to the experimental results

### Bias towards different modal information

It is also valuable to see that MCL-DTI introduces two learnable scalars $\lambda _1$ and $\lambda _2$ to combine the outputs from image modal and text modal information (Eq. 1). This leads to a by-product of MCL-DTI where $\lambda _1$ and $\lambda _2$ actually reflect the model’s bias towards image modal and text modal information.

We explore how different combinations of image modal and text modal affect model performance. We conduct experiments using multiple combinations of methods and summarize the results in Table 5. We perform parallel experiments on Human and Davis datasets and show the learned scalars $\lambda _1$ and $\lambda _2$ from different values. In addition we set a fixed $\lambda _1$ and $\lambda _2$ to observe whether the model has learned the scalars effectively. This observation shows a stable perference for MCL-DTI towards the different design patterns of multimodality. Again the analysis of the data results from the fixed scalars shows that the experimental results all decrease in the absence of a certain modality. We can see that the performance is promising when both scalars are working and both are learnable.

In addition, we consider separately the learning process of the two learnable scalars during the experiment. We conduct experiments to show the learnable parameters $\lambda _1$, $\lambda _2$ from Human and Davis datasets. From the experimental results in Fig. 5, we can see that the learning scalars stabilize in the later stages of the experiment, and the ratio between the two parameters is relatively constant. The variation of the rates in different is relatively small, especially when epoch increases. The ratio of the scalars is inversely proportional to the value of the ROC–AUC, i.e., when the difference between the two learning scalars is greater, the model is less effective. By the fact that the ratios of the final learning scalars are all relatively close, it can be seen that the design pattern in this paper is indeed useful and effective in feature learning of multimodal information.

### Case study

In order to verify the practical ability of the model, we conduct a case study on two highly valuable proteins, namely 3C-like protease (3CLpro) and RNA-dependent RNA polymerase (RdRp). We select experimentally confirmed drug molecules known to interact with them, as well as unrelated drug molecules. The proposed MCL-DTI model was utilized to predict the interaction scores between them. A higher predicted score for interacting drugs and a lower predicted score for unrelated drugs would indicate the practical significance of our proposed model. The experimental results are shown in Table 6.

The 3CLpro is an enzyme found in coronaviruses. 3CLpro plays a crucial role in the replication of the virus by cleaving viral polyproteins into functional proteins necessary for viral assembly and replication. The effectiveness of 3CLpro as a target for antiviral drugs depends on its inhibition. By inhibiting 3CLpro, it is possible to disrupt the replication process of the virus, potentially reducing viral load and slowing down the progression of the disease. RdRp is an enzyme that plays a crucial role in the replication of RNA viruses. RdRp is a target for antiviral drug development, as inhibiting its activity can disrupt viral replication and potentially control viral infections. Therefore, identifying the interaction relationship between drugs and the two aforementioned targets is of great significance. We select 3CLpro and RdRp as the research subjects to determine the reliability of the MCL-DTI model in practical applications by predicting their interactions with candidate drugs such as Baritinib, Sofosbuvir, and Aspirin. Through experiments, we obtain the probability of drug binding to the target.

**Table 6:** Experimental results of 3CLpro and RdRp with candidate drugs

Target	Drug	Predicted Probability	Related or not
3CLpro	Baricitinib	0.999	Related [67]
	Remdesivir	0.998	Related [68]
	Lopinavir	0.995	Related [69]
	Ritonavir	0.874	Related [69]
	Aspirin	0.097	Not Related
RdRp	Sofosbuvir	0.993	Related [70]
	Daclatasvir	0.834	Related [70]
	Lopinavir	0.930	Related [69]
	Ritonavir	0.986	Related [69]
	Aspirin	0.007	Not Related

From the experimental results, we can see that Baricitinib, Remdesivir, Lopinavir, and Ritonavir are highly likely to interact with 3CL pro and Sofosbuvir, Daclatasvir, Lopinavir, Ritonavir are highly likely to interact with RdRp. In fact, this results has been proven by many current studies and clinical trials. On the contrary, the probability of interaction between unrelated drugs aspirin and 3CL pro and RdRp is very low, which is also in line with reality. These experimental results all demonstrate the reliability of MCL-DTI, therefore, we believe that the MCL-DTI model has guiding significance in practical research and drug discovery.

## Conclusion

In this work, we propose a novel model MCL-DTI for DTI task. We exploit for the first time the multimodal information of drugs which characterize them in different modal. We perform semantic learning of molecular image modal and chemical text modal as the embedding representation of the drug by a multi-head self-attentive block. Then, we propose a bi-directional cross-attention mechanism, which allows for deeper semantic learning of drug and target features. From the data results of the experiments, MCL-DTI achieves the best results in all three datasets of DTI, including the balanced datasets and unbalanced datasets. It also explained in the DDI task that MCL-DTI has a strong generalization capability and can be easily applied to other tasks. In additon, ablation experiments further demonstrate the robustness of multimodality and cross-attention block. All the results data indicate that multimodalities and cross-attention learning method can be well applied to DTI or other interaction prediction tasks. In additon, ablation experiments further demonstrate the robustness of multimodality and cross-attention block. All the results data indicate that multimodalities and cross-attention learning method can be well applied to DTI or other interaction prediction tasks. In future work, we consider incorporating other modal information to construct a more rational heterogeneous network. Besides, the effectiveness of deep learning models is still largely limited by the quality and size of the dataset. Therefore, in the next step, we hope to design useful pre-training methods to obtain useful information from large-scale unlabeled biological data in order to further improve the model’s effectiveness.

## Data Availability

The datasets and source codes are publicly available in the GitHub repository, https://github.com/wowowoj/MCL-DTI.git.

## Abbreviations

DTI: Drug–target interaction
MSA: Multi-head self attention
MCA: Multi-head cross attention
ML: Machine learning
GAN: Generative adversarial network
DL: Deep learning
CNN: Convolution neural network
GNN: Graph neural network
DDI: Drug–drug interaction
LN: Layer normalization
SA: Self attention
PE: Positional embedding
